# Differences in nutritional risk assessment between NRS2002, RFH-NPT and LDUST in cirrhotic patients

**DOI:** 10.1038/s41598-023-30031-1

**Published:** 2023-02-27

**Authors:** Peiyan Zhang, Qi Wang, Mengran Zhu, Pingping Li, Yuzhen Wang

**Affiliations:** 1 Department of Geriatric Gastroenterology, Hebei General Hospital, Shijiazhuang, 050000 China; 2 Department of graduate academy, North China University of Science and Technology, Tangshan, 063000 China; 3 Graduate academy, Hebei North University, Zhangjiakou, 075000 China

**Keywords:** Diseases, Gastroenterology

## Abstract

Nutritional status is an independent predictor of outcome in cirrhosis patients. Nutritional Risk Screening 2002 (NRS2002), Royal Free Hospital-Nutritional Prioritizing Tool (RFH-NPT), and Liver Disease Undernutrition Screening Tool (LDUST) were employed to detect cirrhosis with malnutrition risk in this work. Meanwhile, their diagnostic performances were compared to find the best screening method. This work aimed to establish the sarcopenia cut-off value of the transversal psoas thickness index (TPTI), and identify the risk factors for malnutrition. Cirrhosis patients who were admitted to Heibei Gerneral hospital from April 2021 to October 2021 and underwent abdominal CT examination were enrolled. 78 patients were assessed by NRS2002, RFH-NPT, and LDUST. The Global Leadership Initiative for Malnutrition (GLIM) criteria were selected as the gold standard for the diagnosis of malnutrition. Meanwhile the cut-off value of sarcopenia was established based on the TPTI of malnourished patients. Logistic regression analysis was adopted to assess the influencing factors of malnutrition risk and malnutrition. The prevalence of malnutrition was 42.31%. The prevalence of malnutrition risk was 32.1%, 61.5%, and 62.8% with NRS2002, RFH-NPT, and LDUST, respectively. NRS2002 presented the best specificity compared with the other methods, while RFH-NPT showed the highest sensitivity. The optimal gender-specific TPTI cut-off value for diagnosing sarcopenia was determined as TPTI < 14.56 mm/m (male) and TPTI < 8.34 mm/m (female). In the multivariate analysis, ascites was associated with malnutrition risk, while sarcopenia showed a significant risk for malnutrition. NRS2002 and RFH-NPT were superior to LDUST at detecting the malnutrition in cirrhosis patients diagnosed according to GLIM criteria. The gender-specific TPTI cut-off value was TPTI < 14.56 mm/m (male) and TPTI < 8.34 mm/m (female). Malnutrition risk should be screened for patients with ascites as soon as possible. In addition, it was important to evaluate malnutrition in sarcopenia patients in time.

## Introduction

Malnutrition is a common complication of cirrhosis. It can prolong the hospital stays, especially the time in intensive care unit (ICU), accelerate the decompensation, and increase mortality^1^. Early detection and diagnosis are vitally associate to prognosis of cirrhosis patients^2^.

However, nutrition assessment and nutrition screening are challenges to this group of patients in China. It is caused by multiple factors, including the absence of gold standard for nutrition assessment, unavailable validated screening tools, and few studies research on the difference of screening tools.

The prevalence of malnutrition in cirrhosis ranges from 5 to 99%, depending on the severity of liver cirrhosis and the diagnostic criteria^3–5^. Multiple scientific nutrition societies have established the GLIM criteria as a global diagnostic reference for malnutrition^6^. As far as we know, the GLIM criteria for cirrhosis have been investigated in some studies.

Various malnutrition risk screening tools have been proposed with different advantages and disadvantages, but their results vary among different studies. NRS2002^7^ is most widely used in the world due to some limitations in the application of cirrhosis because of the absence of ascites parameters. RFH-NPT^8^ andLDUST^9^ are established for patients with liver cirrhosis, while their data in China are few.

Cirrhosis is complicated by sarcopenia^10^. TPTI is an easy, reliable, and repeatable parameter for assessing the sarcopenia since it is computed tomography (CT)-based and just needs to measure the psoas diameter^11^. However, data on TPTI in cirrhosis are still lacking in China and the cut-off value of sarcopenia is rare.

In this work, the GLIM was undertake as the gold standard to diagnose malnutrition and three malnutrition risk screening tools (NRS2002, RFH-NPT and LDUST) were evaluated. This work aimed to compare the differences in these 3 tools and identify their superiorities in live cirrhosis. Meanwhile, the best cut-off value of sarcopenia was identified by comparing the TPTI in nutrition group and malnutrition group.

## Material and methods

This work included 78 cirrhosis patients who were admitted to Heibei General Hospital from April 2021 to October 2021 and underwent abdominal CT examination. All patients met the diagnostic criteria of the ‘Chinese Guidelines on the Management of Liver Cirrhosis’. Patients with any of below conditions had to be excluded: severe cardiopulmonary disease, cancer, inflammatory bowel disease, and other metabolic disorders. In addition patients over 80 and under 18 years old were excluded.

This work was approved by the ethics committee of the Heibei General Hospital (2021-267) and was performed in accordance with the Declaration of Helsinki. Written informed consents were obtained from all the participants prior to the enrollment.

The data included gender, age, height, body weight, body mass index (BMI), routine blood examination, biochemical parameters and other laboratory tests. The body weight was adjusted according to water content retention. The weight was reduced by 5%,10%, and 15% in mild, moderate, and severe ascites, respectively and it was reduced by 5% in patients who had combined peripheral edema. The classical nutritional markers included calf circumference, arm circumference, and arm muscle circumference.

The abdominal CT images of all subjects were obtained within 2 weeks. The axial psoas muscle thickness (APMT) was defined as the largest antero-posterior diameter of the right psoas muscle at the L3 level and recorded in millimeters(mm), and the longest transverse diameter which was perpendicular to APMT was defined as the psoas muscle transversal psoas muscle thickness (TPMT), TPTI = TPMT / height (mm/m)^12^ (Appendix 1).

The NRS2002, RFH-NPT, and LDUST were used for malnutrition risk screening, and the GLIM criteria were selected for the assessment of malnutrition. Patients who were assessed to be at risk of malnutrition were included in the risk group. According to the GLIM criteria, the 78 patients were divided into a malnutrition group and a nutrition group. All tools were performed by an experienced clinical physician.

The NRS2002 contained nutritional impairment scores, severity of disease scores, and an age adjustment in which over 70 years adds one point^13^. Nutritional impairment ranged from 1 to 3 according to the weight loss over 5% in 3 to 1 months or the food intake reduced < 50%, 50–75% and > 75% compared with the values in previous week. It can be recorded as 3 points if the BMI < 18.5 kg/m^2^. The severity of disease was assessed as 1, 2, and 3 points, respectively, in different situations. Cirrhosis was recorded as 1 point. The total score of three parts classified patients into a no risk (< 3 points) and a malnutrition risk (≥ 3 points) group.

RFH-NPT was performed in three steps^14^. First, the alcoholic hepatitis patients or those who needed tube feeding were assessed as high risk. Those who failed to meet the conditions above needed to assess ascites or edema and its impacts on food intake and weight loss. If ascites or edema was found, 1 point was assigned. The food intake impact on ascites or edema was recorded as 0 to 2 points, which corresponded to no influence, occasional influent, and influent. If the food intake was reduced by half in 5 days, 2 points were given, or otherwise 0 points were assigned. The weight loss in the past 3–6 months ranged from 0 to 2 points, which meant for no, being unable to evaluate, and yes, respectively. Those who did not have fluid overload were estimated using BMI, unplanned weight loss, and dietary intake. BMI was recorded as 0 to 2 points according to > 20 kg/m^2^, 18.5–20 kg/m^2^ and  < 18.5 kg/m^2^, respectively. The unplanned weight loss in the past 3–6 months ranged from 0 to 2 points according to < 5%, 5–10%, and  > 10%, respectively. It should assess whether the patient is in acute exacerbation and has or may have no nutritional intake for more than 5 days. It was recorded as 2 points if yes. Patients with a score of 2–7, were at high risk, and they were at low risk with a score of 0 and moderate risk with a score of 1.

The LDUST included six questions. Food intake, weight loss, muscle loss, swelling or fluid, and daily activities were determined as grades A, B and C^15^. 5 or more points were included in grade A, which was identified as no risk, while 2–5 points in grade B and  < 2 points in grade C were identified as malnutrition risk.

There were two components in the GLIM criteria: phenotypic criterion and etiologic criterion^6^. Phenotypic criteria included weight loss, low BMI, and reduced muscle mass, while etiologic criterion contained reduced food intake or assimilation and inflammation. All the cirrhosis patients were considered to present chronic disease-related inflammation, so they all met the etiologic criterion. Weight loss which went down 5% in 6 months or 10% over 6 months was assessed in this work. Meanwhile, the low BMI (BMI < 18.5 kg/m^2^ for patients < 70 years old or BMI < 20 kg/m^2^ for patients > 70 years old) was assessed. If the weight and BMI were in normal ranges, their calf circumference can be assessed to check whether the muscle mass was reduced. According to a Japanese standard, reduced muscle mass was assessed by calf circumference ≤ 30 cm (male) and ≤ 29 cm (female) since there was no sarcopenia standard in China^16^. The patients who met one phenotypic criterion and one etiologic criterion were diagnosed with malnutrition in the GLIM criteria.

The TPTI of the malnutrition group based on the GLIM criteria was employed to establish the cut-off value of sarcopenia in cirrhosis.

### Statistical analysis

Data were analyzed using SPSS 25 and Graphpad Prism 8.3.0. Data that which conformed to the normal distribution were expressed as the mean ± standard deviation (SD). The comparison between the two groups was performed by using the t test. Non-normally distributed data were represented by Median (P25 ~ P75) and the comparison between two groups was performed using the Mann–Whitney U test. The count data was expressed as percentages (%), and the two groups were compared by the Chi-square test. Correlation and consistency were assessed by spearman correlation coefficient analysis and Kappa consistency test, respectively. Receiver operating characteristic (ROC) curves were adopted to evaluate the ability of the three screening tools to distinguish the malnourished patients. The GLIM criteria were selected as the reference, ROC curves were generated for TPTI, and the best cut-off values to diagnose sarcopenia were determined using the Youden Index. Sensitivity, specificity, positive predictive value (PPV), negative predictive value (NPV), positive likelihood ratio (PLR), and negative likelihood ratio (NLR) were estimated to test the quality of screening tools to predict malnutrition. Logistic regression analysis was employed to assess the influencing factors of malnutrition risk, malnutrition, and sarcopenia. P value less than 0.05 meant that the difference was significant.

## Results

### Characteristics of patients

The characteristics of the 78 included subjects were detailed in Table 1. The mean age was 54.5 ± 12.54 years old, and 50 (64.1%) of the patients were male. The most frequent etiology of cirrhosis was hepatitis B virus (HBV) (43.6%), followed by alcohol (24.4%). Based on the stage of the disease, 82.1% of patients was decompensated, 44.9% of patients had Child-A, 38.5% had Child-B, and 16.7% had Child-C. According to the GLIM criteria, malnutrition was diagnosed in 33 patients (42.3%). Comparison between the malnutrition group and the nutrition group revealed that there were significant differences in TPTI, arm circumference, NRS2002, RFH-NPT, and LDUST. Malnourished patients were observed with reduced TPTI and arm circumference.

**Table 1:** Baseline data of total patients and per nutritional state, stratified by GLIM criteria.

Characteristics	Total (n = 78)	Malnutrition (n = 33)	No malnutrition (n = 45)	P-value
Age	54.50 ± 12.54	51.60 ± 12.27	56.62 ± 12.43	0.081
Gender, n (%)				
Male	50 (64.1%)	24 (72.7%)	26 (57.8%)	0.174
Weight (Kg)	67.28 ± 14.02	65.47 ± 15.62	68.60 ± 12.74	0.332
BMI (Kg/m^2^)	24.64 ± 4.36	23.66 ± 4.98	25.36 ± 3.74	0.088
Etiology, n (%)				
HBV	34 (43.6%)	16 (48.5%)	18 (40.0%)	0.677
Alcohol	19 (24.4%)	10 (30.3%)	9 (20.0%)	
HCV	4 (5.1%)	1 (3.0%)	3 (6.7%)	
Cholestatic	7 (9.0%)	3 (9.1%)	4 (8.9%)	
MAFLD	6 (7.7%)	1 (3.0%)	5 (11.1%)	
Drug	1 (1.3%)	0 (0%)	1 (2.2%)	
Unknown	7 (9.0%)	2 (6.1%)	5 (11.1%)	
Decompensated, n (%)	64 (82.1%)	25 (75.8%)	39 (86.7%)	0.244
Ascites, n (%)	31 (39.7%)	15 (45.5%)	16 (35.6%)	0.483
MELD sore	10.08 ± 5.13	10.55 ± 5.02	9.74 ± 5.24	0.495
Child–Pugh, n (%)				
Child-A/B	64 (82.1%)	23 (69.7%)	41 (91.11%)	0.019
Child-C	14 (17.9%)	10 (30.3%)	4 (8.89%)	
TPTI (mm/m)	14.15 ± 4.41	12.98 ± 4.15	15.00 ± 4.45	0.045
Arm circumference (cm)	27.71 ± 4.24	26.55 ± 4.38	28.56 ± 3.96	0.037
Tricipital skinfold thickness (cm)	1.25 (0.85–1.85)	1.05 (0.66–1.75)	1.45 (0.95–1.95)	0.051
Arm muscle circumference (cm)	23.27 ± 3.58	22.73 ± 3.38	23.88 (20.94–25.88)	0.255
Calf circumference (cm)	34.63 ± 4.13	34.12 ± 4.90	35.00 ± 3.47	0.382
Total protein (g/L)	65.25 (59.70–70.78)	63.86 ± 11.46	65.00 ± 9.45	0.634
Albumin (g/L)	34.40 (27.15–38.55)	32.01 ± 7.11	33.96 ± 6.71	0.220
Prealbumin (g/L)	9.70 (6.83–13.53)	9.00 (6.05–11.90)	10.83 ± 4.12	0.077
LDUST, n (%)				
No risk	29 (37.2%)	5 (15.2%)	24 (53.3%)	< 0.001
Risk	49 (62.8%)	28 (84.8%)	21 (46.7%)	
RFH-NPT, n (%)				
No risk	30 (38.5%)	3 (9.1%)	27 (60.0%)	< 0.001
Risk	48 (61.5%)	30 (90.9%)	18 (40.0%)	
NRS2002, n (%)				
No risk	53 (67.9%)	11 (33.3%)	42 (93.3%)	< 0.001
Risk	25 (32.1%)	22 (66.7%)	3 (6.7%)	

*BMI* body mass index, *HBV* hepatitis B virus, *HCV* hepatitis C virus, *MAFLD* metabolic dysfunction-associated fatty liver disease, *MELD* the model for end-stage liver disease, *TPTI* transversal psoas thickness index, *LDUST* liver disease undernutrition screening tool, *RFH-NPT* royal free hospital-nutritional prioritizing tool, *NRS2002* nutritional risk screening 2002.

### Comparison between screening tools and GLIM criteria

In this work, NRS2002 exhibited the highest correlation with the GLIM criteria based on spearman correlation coefficient (Spearman’s r value of 0.635), followed by RFH-NPT and GLIM criteria (Spearman’s r value of 0.517) (Table 2). Besides, the highest consistency was observed between the NRS2002 and GLIM criteria based on the Kappa consistency test (Kappa = 0.62) (Table 3).

**Table 2:** Spearman correlation coefficients of different tools.

	NRS2002	RFH-NPT	LDUST	GLIM
NRS2002	1	0.486	0.415	0.635
RFH-NPT	0.486	1	0.373	0.517
LDUST	0.415	0.373	1	0.390
GLIM	0.635	0.517	0.390	1

*NRS2002* nutritional risk screening 2002, *RFH-NPT* royal free hospital-nutritional prioritizing tool, *LDUST* liver disease undernutrition screening tool, *GLIM* global leadership initiative for malnutrition.

**Table 3:** Kappa coefficients of different tools.

	NRS2002	RFH-NPT	LDUST	GLIM
NRS2002	1.00	0.41	0.34	0.62
RFH-NPT	0.41	1.00	0.37	0.48
LDUST	0.34	0.37	1.00	0.36
GLIM	0.62	0.48	0.36	1.00

*NRS2002* nutritional risk screening 2002, *RFH-NPT* royal free hospital-nutritional prioritizing tool, *LDUST* liver disease undernutrition screening tool, *GLIM* global leadership initiative for malnutrition.

### Validation of screening tools

**Figure 1:**
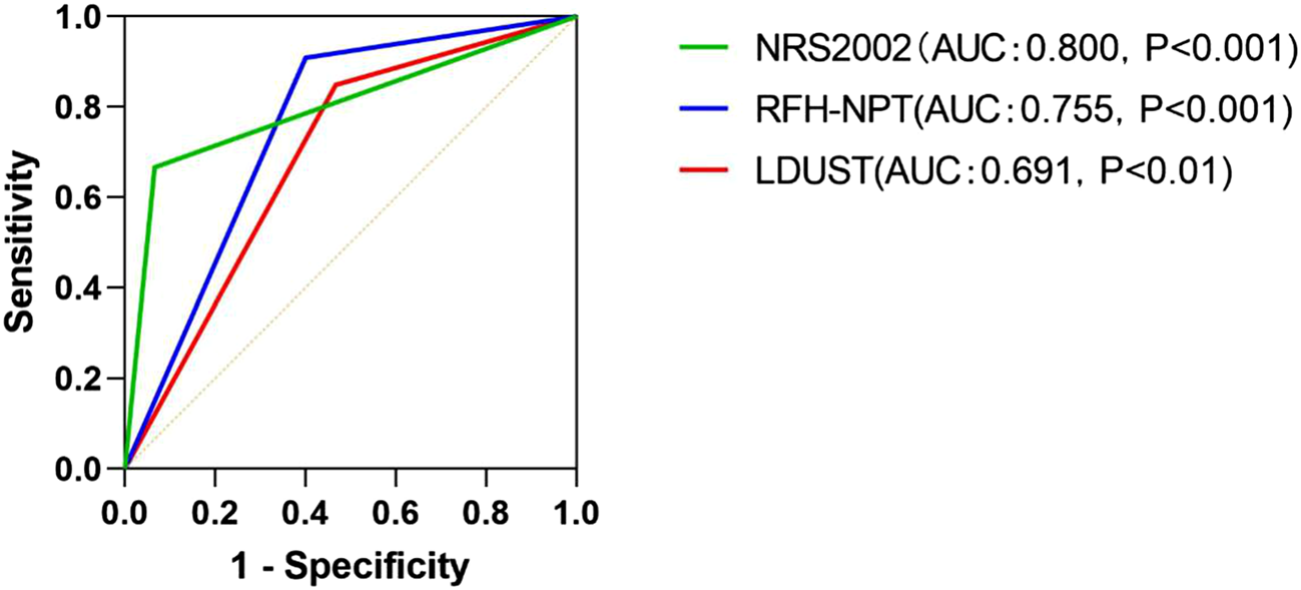
ROC curves of the screening tools for the diagnosis of malnutrition using GLIM criteria as a benchmark.

Compared to the GLIM criteria as the benchmark for malnutrition diagnosis, NRS2002 presented the highest accuracy in detecting malnutrition based on the area under the ROC curve (AUC), followed by RFH-NPT (AUC, 0.800 and AUC, 0.755, respectively) (Fig. 1, Table 4). RFH-NPT showed the highest sensitivity (90.91%) and the lowest specificity (60.00%), while NRS2002 presented the highest specificity (93.33%) and the lowest sensitivity (66.67%). LDUST showed a moderate sensitivity and the lowest specificity. It was noticeable that the accuracy of NRS2002 and RFH-NPT was higher than that of LDUST.

**Table 4:** Measures of diagnostic validity of screening tools.

Validity criteria	NRS2002	RFH-NPT	LDUST
AUC (95% CI)	0.800 (0.692–0.908)	0.755 (0.646–0.863)	0.691 (0.573–0.809)
Accuracy, %	82.05	73.08	66.67
Sensitivity, %	66.67	90.91	84.85
Specificity, %	93.33	60.00	53.33
Youden index	0.60	0.51	0.38
PPV, %	88.00	62.50	57.14
NPV, %	79.25	90.00	82.76
PLR	10.00	2.27	1.82
NLR	0.36	0.15	0.28

*AUC* the area under the ROC curves, *PPV* positive predictive value, *NPV* negative predictive value, *PLR* positive likelihood ratio, *NLR* negative likelihood ratio, *NRS2002* nutritional risk screening 2002, *RFH-NPT* royal free hospital-nutritional prioritizing tool, *LDUST* liver disease undernutrition screening tool.

### Defining cut-off values for sarcopenia that correlate with malnutrition

**Figure 2:**
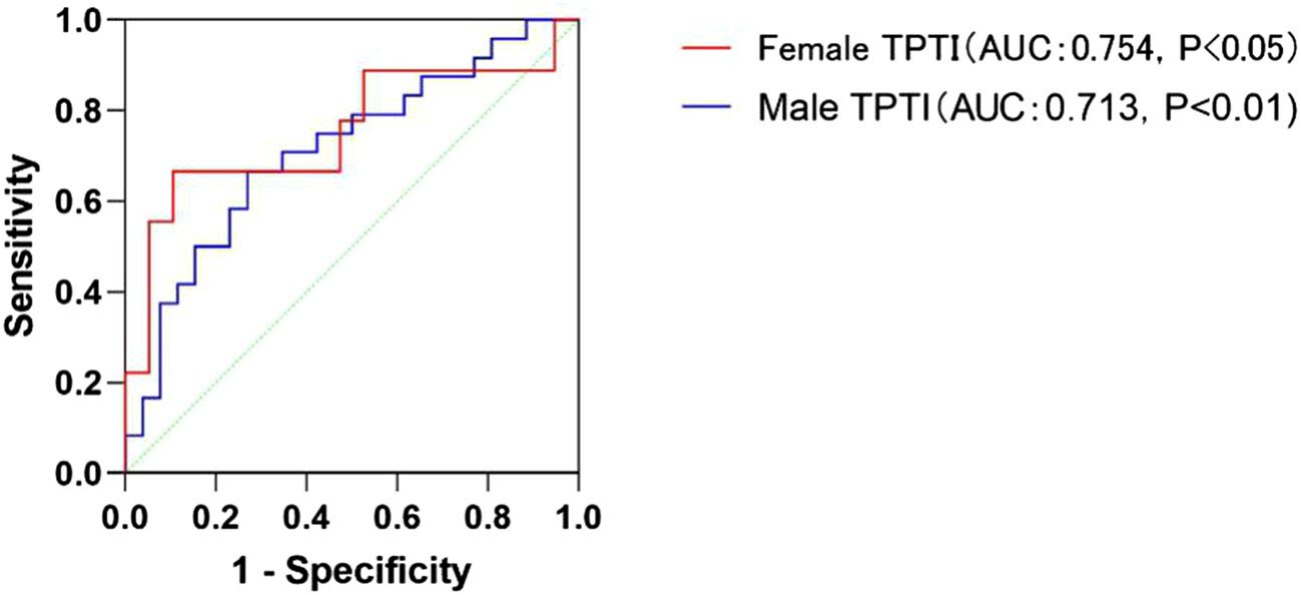
Predictive results of TPTI on the diagnosis of malnutrition using GLIM criteria as a benchmark.

Using GLIM criteria as the reference, the cut-off values were calculated and derived from ROC curves and the Youden index (Fig. 2). The TPTI-AUC was 0.713 (0.569–0.858, P < 0.01) for men, and 0.754 (0.533–0.976, P < 0.05) for women. Cut-off values for sarcopenia were TPTI < 14.56 mm/m (male) and < 8.34 mm/m (female). With the threshold of sarcopenia, sarcopenia was diagnosed in 31(39.74%) cirrhosis patients, of which 22 (70.97%) were malnourished and 9 (29.03%) were not malnourished (X^2^ = 17.313, P < 0.001). Regardless of gender, the rate of sarcopenia in the malnutrition group was significantly higher than that in the nutrition group (P < 0.01). The incidence showed no differences in Child-A, Child-B, and Child-C, and gender.

### Logistic regression analyses for malnutrition risk and malnutrition

The logistic regression analyses revealed that albumin, prealbumin, lactate dehydrogenase, alkaline phosphatase, ascites, and sarcopenia were influencing factors for malnutrition risk. Among them, only ascites was an independent risk factor (Table 5). TPTI and sarcopenia were the interfering factors to malnutrition, while only sarcopenia was an independent factor, which was revealed by the multivariate analyses (Table 6).

**Table 5:** Univariate and multivariate logistic regression analyses for malnutrition risk.

Parameter	Univariate logistic regression			Multivariate logistic regression		
	OR	95% CI	P-value	OR	95% CI	P-value
Albumin (g/L)	0.898	0.821–0.982	< 0.05			
Prealbumin (g/L)	0.835	0.729–0.955	< 0.05			
Lactate dehydrogenase (U/L)	1.011	1.001–1.021	< 0.05			
Alkaline phosphatase (U/L)	1.019	1.003–1.035	< 0.05			
Ascites	4.375	1.146–16.697	< 0.05	5.665	1.284–24.994	< 0.05
Sarcopenia	4.375	1.146–16.697	< 0.05

**Table 6:** Univariate and multivariate logistic regression analyses for malnutrition.

Parameter	Univariate logistic regression			Multivariate logistic regression		
	OR	95% CI	P-value	OR	95% CI	P-value
TPTI (mm/m)	0.88	0.778–0.989	< 0.05			
Sarcopenia	8.00	2.861–22.370	< 0.001	8.739	2.543–30.038	< 0.001

*TPTI* transversal psoas thickness index.

## Discussion

There are various malnutrition methods to assess cirrhosis, such as SGA and RFH-SGA. These two methods are time-consuming and poorly applied in China. In 2018, the global nutrition societies established the GLIM criteria to form a global consensus on the diagnosis of malnutrition. After that, the use of GLIM criteria was in many crowds, such as cancer or older adults^17,18^.

At present, few studies have examined on the application of the GLIM criteria for malnutrition in cirrhosis. In addition, some studies have found that compared with SGA and RFH-GA, GLIM can better predict the mortality of patients with chronic liver disease^19,20^. Meanwhile, there are increasingly more studies selecting the GLIM standard as the gold standard in patients with liver cirrhosis^5,21^.

Boulhosa RSSB and Diego were the first batch researchers to focus on this field^5,22^. With the use of GLIM criteria, 42.3% of cirrhosis patients were identified to be complicated with malnutrition in this work, which is similar to data from Boulhosa RSSB and Diego (57.2% and 38.1%, respectively)^5,22^.

The assessment using the GLIM criteria is performed in two steps. First, whether patients were at risk of malnutrition should be assessed using nutritional screening tools. Second, the GLIM criteria are adopted to assess the malnutrition risk patients with malnutrition. This work aimed to determine which nutritional screening tools could obtain findings consistent findings with the results of the GLIM criteria, so that they could be applied in the first step of Glim in the follow-up clinical work. This work validated three screening tools in predicting malnutrition when diagnosed based on the GLIM criteria.

NRS2002, RFH-NPT, and LDUST were validated and effective methods for nutritional screening of patients with liver cirrhosis. The GLIM group recommend the NRS2002, but this work also found that the RFH-NPT showed the same correlation and consistency as NRS2002.

This work confirmed that NRS2002 and RFH-NPT were the optimal tools applied in patients with the best specificity and sensitivity, respectively. This conduction is in line with previous studies that revealed superiorities of NRS2002 and RFH-NPT in specificity and sensitivity^23,24^.

Compared with NRS2002, GLIM takes BMI, liver cirrhosis, and food intake as evaluation items. The difference is that the BMI of GLIM is more detailed according to patients’ ages, unlike NRS2002, where is the BMI of all patients is 18.5 kg/m^2^ as a reference. In terms of food intake, the NRS2002 pays more attention to changes in a short time (within 1 week), while the GLIM expands the range to changes within two weeks. Besides, the NRS2002 assesses the weight loss within 1–3 months, while the GLIM only evaluates weight loss within 6 months of malnutrition grading. There are so many similarities between them that the good consistency between them is easy to understand.

RFH-NPT focuses on fluid retention, and patients are divided into two groups according to different results, and receive different assessment steps for BMI and weight loss are carried out. GLIM is proposed for patients with various diseases. It is not included in the item of fluid retention, but in the assessment of muscle loss. For patients with liver cirrhosis, the assessment of muscle loss is more objective and accurate than fluid retention.

LSUST is a subjective evaluation form for patients. Many Chinese patients will ignore their weight, muscle loss, and diet changes, so the consistency between LSUST and GLIM is not as good as that of the other two methods.

NRS2002 score needs over 3 when it is considered as malnutrition risk, RFH-NPT score reaches 1 point, while LDUST needs two of the six subjective questions to get B or C. NRS2002 is relatively more rigid to confirm the diagnose malnutrition risk. Therefore, patients who were evaluated as having no malnutrition risk by NRS2002 can be more accurately diagnosed as having no malnutrition. RFH-NPT considers patients with ascites or peripheral edema as malnutrition risk, while LDUST needs to merge the fluid overload with another entry. NRS2002 does not involve fluid retention. This is the reason that RFH-NPT shows the highest sensitivity. Among the three methods, NRS2002 and RFH-NPT can be better applied to screen the malnutrition risk in cirrhosis, while LDUST is more subjective and can be used for self-screening management in a short time, so it is applicable to clinical practice.

TPTI is a simple and rapid method of diagnosing sarcopenia. In contrast with the L3 skeletal muscle index which is commonly used abroad^25,26^, TPTI can be measured by abdominal CT but requires no special measurement software. This work found the cut-off value of sarcopenia as TPTI < 14.56 mm/m (male) and < 8.34 mm/m (female), which was higher than the value mentioned in a foreign study^27^, which may be related to the distinct way to get thecut off values. Psternosrto^27^ obtained his threshold by the mortality of patients in a follow-up period, but the malnutrition of patients was adopted in this work. The average TPTI of deaths was lower than that of malnourished patients. The threshold in men is higher than that in women, which is similar all over the world, because of the influence of gender hormones that men have more muscle groups. Even after adjustment for height, the amount of psoas muscle still shows a significant gender difference^28^.

Because nutritional status is an intervention that can impact prognosis, it is expect to identify the risk factors for different nutritional stages and to take patients with risk factors seriously. The data in this work suggested that the malnutrition risk of patients with ascites was 5.665 times as that of patients without ascites. Patients with ascites are more likely to suffer from anorexia and induced intake due to severe gastrointestinal symptoms and early satiety^29,30^. In addition, rest energy expenditure increases significantly in these patients^31^. The nutrition assessment for most ascites patients with increased weight is often ignored by clinicians. According to the literature, Xiaoyu Wang and their colleagues used RFH-NPT to assess 135 cirrhosis patients and found that ascites increased the malnutrition risk, which was similar to the findings of this work^32^. Malnutrition can accelerate the progression of ascites and significantly increase the probability of refractory ascites^33,34^. However, ascites was not proven to be a risk factor for malnutrition in this work, which may be because of the small sample size and the use of GLIM criteria as a standard for malnutrition assessment.

The impact factors of sarcopenia have been studied for many years. Studies have shown that Child–Pugh, arm circumference, arm muscle circumference, age, myostatin, albumin, and prealbumin are the impact factors^35–37^. The research of Hsu CS found that the prevalence of sarcopenia increased in men and patients with ascites, liver failure, and kidney failure^38^. A similar conclusion was reached by this work, in which prealbumin was an independent factor.

On the other hand, there were some limitations in this work. First, it may be subject to population bias due to the small sample size and single center study may cause population bias. Second, the effects of nutritional support therapy on the prognosis of patients with malnutrition or sarcopenia have not been studied.

## Conclusion

As far as we know, this work was the first study in China to compare the NRS2002, RFH-NPT, and LDUST with GLIM criteria in patients with cirrhosis. In the light of GLIM criteria, the prevalence of malnutrition was 42.3%, which confirmed that NRS2002 and RFH-NPT could better screen for malnutrition risk in clinical application in patients with cirrhosis. Moreover, TPTI < 14.56 mm/m (male) and < 8.34 mm/m (female) were proven to be the cut-off values of male and female patients with sarcopenia, respectively. In conclusion, this work verified that clinicians should attach importance to the patients with ascites and sarcopenia. The datasets generated or analyzed in this work were available from the corresponding author on reasonable request.

## Supplementary Information

Supplementary Information.
