# Supplementary material for: Differences in nutritional risk assessment between NRS2002, RFH-NPT and LDUST in cirrhotic patients
Source: Sci Rep. 2023 Feb 27;13:3306. doi: 10.1038/s41598-023-30031-1 (PMC9971362; doi:10.1038/s41598-023-30031-1)
Supplement: Supplementary file 1 — Supplementary Information. [file 41598_2023_30031_MOESM1_ESM.docx]

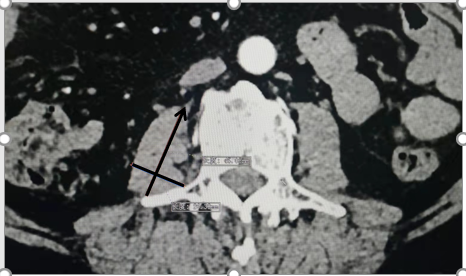


Appendix 1: Measurement of APMT and TPMT.

Arrow: axial psoas muscle thickness (APMT)

Straight line: the psoas muscle transversal psoas muscle thickness (TPMT).
